# Nitrite producing bacteria inhibit reinforcement bar corrosion in cementitious materials

**DOI:** 10.1038/s41598-018-32463-6

**Published:** 2018-09-20

**Authors:** Yusuf Çağatay Erşan, Kim Van Tittelboom, Nico Boon, Nele De Belie

**Affiliations:** 1 Magnel Laboratory for Concrete Research, Faculty of Engineering and Architecture, Ghent University, B-9052 Ghent, Belgium; 2 Centre for Microbial Ecology and Technology (CMET), Faculty of Bioscience Engineering, Ghent University, B-9000 Ghent, Belgium

## Abstract

Chemicals and synthetic coatings are widely used to protect steel against corrosion. Bio-based corrosion inhibition strategies can be an alternative in the arising bioeconomy era. To maintain the good state of steel reinforcement in cracked concrete, microbe-based self-healing cementitious composites (MSCC) have been developed. Yet, proposed strategies involve reasonably slow crack filling by biomineralization and thus risk the possible rebar corrosion during crack healing. Here we upgrade the rebar protection to a higher level by combining MSCC with microbial induced corrosion inhibition. Presented NO_3_^−^ reducing bacterial granules inhibit rebar corrosion by producing the anodic corrosion inhibitor NO_2_^−^ and meanwhile heal a 300-µm-wide crack in 28 days. During 120 days exposure to 0.5 M Cl^−^ solution, the rebars in cracked MSCC keep showing open circuit potentials above the critical value of −250 mV and they lose less than 2% of the total rebar material which corresponds to half the material loss in cracked plain mortar. Overall, the obtained rebar protection performance is comparable with that of uncracked mortar and mortar containing chemical inhibitor, hence the microbe-based system becomes an alternative to the traditional methods.

## Introduction

Microcracks in reinforced concrete structures, especially in marine environment, facilitate the ingress of substances such as Cl^−^, SO_4_^2−^, CO_2_ and O_2_ towards the reinforcement bar (rebar). Hence, the steel passivation may quickly be lost and rebar corrosion starts which results in further durability issues and ultimately structural failure. Conventionally used coatings and corrosion inhibitors are often expensive and they may contain toxic volatile organic solvents, causing severe environmental concerns^1–3^. The construction sector needs effective, cost-efficient, sustainable and green corrosion control methods to deal with durability issues.

Microbial induced calcite precipitation (Eqs (1 and 2)) enables development of microbe-based self-healing cementitious composites (MSCC) that can autonomously heal their microcracks^4–10^. So far, regardless of the proposed metabolic pathway, MSCC perform better than conventional mortars in terms of water tightness regain^7,9,10^. Such regain in functionality is desired to hinder the ingress of aforementioned aggressive substances and to protect the good state of the rebar^11–13^. In previous microbe-based self-healing studies, it was claimed that biomineralization in microcracks is an effective method for protection of the steel reinforcement^4–6,9,14^. Yet, available data lacks solid evidence on the protective effect of MSCC against rebar corrosion. Especially the corrosion behaviour of steel reinforcement in cracked and uncracked MSCC is unknown.

The biochemical crack healing process requires a time span of 28 to 70 days for immersed mortar specimens^4,5^. We speculate that rebars are still susceptible to attack by aggressive ions during the reported time period of biochemical crack healing due to the fact that the water tightness could be regained effectively only after complete healing of the crack. Therefore, especially for applications of MSCC in marine environment, complementary mechanisms that provide corrosion inhibition are necessary to effectively protect rebars from corrosion during microbe-mediated healing of cracks.

Among the reported microbial pathways, microbial nitrate reduction can be more advantageous on this pursuit compared to the others (urea hydrolysis and aerobic respiration) as NO_3_^−^ reducing microorganisms simultaneously produce NO_2_^−^, a commercially used anodic corrosion inhibitor, as an intermediate product^2,15^, and induce bio-mineralization through the following reactions.

1 $${\rm{HCOOH}}+{{\rm{NO}}}_{3}^{-}\,\mathop{\longrightarrow }\limits^{nitrate\,reductase}\,{{\rm{CO}}}_{2}+{{\rm{H}}}_{2}{\rm{O}}+{{\rm{NO}}}_{2}^{-}$$

2 $${{\rm{Ca}}}^{2+}+{{\rm{CO}}}_{2}+{{\rm{H}}}_{2}{\rm{O}}\to {{\rm{CaCO}}}_{3}+2{{\rm{H}}}^{+}$$

The major role of NO_2_^−^ in steel corrosion inhibition is to interfere with the migration of the ferrous ions from the anodic zones of the rebar. Ferrous hydroxide formed in anodic reactions is rapidly stabilized by the NO_2_^−^ ions in the form of ferric oxide (Eq. (3)). Therefore, the formation of mobile iron chloride complexes is hindered. Moreover, the precipitated ferric oxides create another passive layer which acts as a barrier against further corrosion and material loss. Since NO_2_^−^ interferes with the reaction between ferrous hydroxide and chloride ions, the NO_2_^−^ concentration in the environment is crucial. Corrosion inhibition studies where chemical NO_2_^−^ was used as corrosion inhibitor revealed that a [NO_2_^−^]:[Cl^−^] ratio of 0.34 to 1.00 is enough for optimum corrosion inhibition^2,15,16^.

3 $$2{{\rm{F}}{\rm{e}}}^{2+}+2{{\rm{O}}{\rm{H}}}^{-}+2{{\rm{N}}{\rm{O}}}_{2}^{-}\to 2{\rm{N}}{\rm{O}}+{{\rm{F}}{\rm{e}}}_{2}{{\rm{O}}}_{3}+{{\rm{H}}}_{2}{\rm{O}}$$

Our team previously developed a non-axenic granulated NO_3_^−^ reducing microbial culture named as ‘activated compact denitrifying core’ (ACDC), which reveals satisfactory distribution and stability in mortar and effectively promotes self-healing of cracks^7,17,18^. Using the same culture, our initial attempts to explore the influence of microbially produced NO_2_^−^ on steel plates revealed the possibility of microbial induced corrosion inhibition in weak salt solutions (0.05 M Cl^−^) (ref.^17^). The findings revealed that the microbial produced NO_2_^−^ started to be effective when the [NO_2_^−^]:[Cl^−^] ratio exceeded 0.50. Yet, the investigated test conditions provided limited information which was not enough to demonstrate the corrosion behaviour of reinforced MSCC containing ACDC in marine environment. First of all, in marine environment, typical Cl^−^ concentration is around 0.50 M which is 10 times higher than the concentration tested in our previous study and the ionic activity (µ) of the seawater is around 0.70 (refs^19,20^). Either of these variables may adversely affect the activity of microorganisms^21^ and thus their influence on the activity of in-house developed ACDC granules should be investigated. Secondly, the effect of the mortar matrix was not included in the reported corrosion tests where steel plates were directly tested in a corrosive nutrient solution. Since the alkalinity of mortar can passivate the steel with a thin oxide layer, the corrosion behaviour of a rebar can be better understood only if the steel is tested while embedded in mortar. Finally, the corrosion was induced by polarization experiments rather than the natural disruption of the passive film and further corrosion of the steel in long-term. Therefore, the influence of biomineralization on corrosion behaviour of the rebar was yet unknown. Due to the limitations in the experimental design of the preliminary study, the actual rebar corrosion inhibition performance of ACDC in a cementitious composite exposed to marine conditions still remains unclear. Therefore, our main objective was to evaluate the rebar protection performance of ACDC containing MSCC in corrosive marine conditions during and following the crack healing period. The study was conducted in two consecutive steps; (i) pre-assessment of the ACDC culture for salt tolerance by testing resuscitation and microbial activity of ACDC culture under a variety of ionic strengths (*µ*), (ii) investigation of the corrosion behaviour of rebars embedded in MSCC in simulated marine conditions.

## Results

### Culture tolerance to possible osmotic stress in marine environment

A typical seawater (*µ* ~ 0.7 and [Cl^−^] ~ 0.5 M) and gradual accumulation of non-metabolised ions that leach from the mortar (such as OH^**−**^, Na^+^, Ca^2+^ and Fe^2+^, among others) are expected *in situ*. Therefore, the effects of the most relevant ions (Cl^−^ and NO_3_^−^) on resuscitation and microbial activity of NO_3_^−^ reducing ACDC culture were tested (Table 1).

**Table 1:** Microbial activity of ACDC at various Cl^−^ − NO_3_^−^ combinations and ionic strengths (µ).

*µ* (M)	Cl^−^ (M)	NO_3_^−^ (M)	HCOO^−^ (M)	Na^+^ (M)	NO_3_^−^ reduction rate^a^ (mmol.g^−1^.d^−1^)	NO_2_^−^ accumulation rate^a^ (mmol.g^−1^.d^−1^)
0.70	0.30	0.20	0.20	0.70	2.70 ± 0.01	2.30 ± 0.25
0.80	0.30	0.30	0.20	0.80	1.40 ± 0.15	1.00 ± 0.15
	0.40	0.20	0.20	0.80	1.00 ± 0.10	0.90 ± 0.15
0.90	0.40	0.30	0.20	0.90	0.55 ± 0.25	0.40 ± 0.20
	0.50	0.20	0.20	0.90	0.80 ± 0.20	0.60 ± 0.15
1.00	0.40	0.40	0.20	1.00	0.80 ± 0.05	0.65 ± 0.10
	0.50	0.30	0.20	1.00	0.70 ± 0.05	0.50 ± 0.20
1.10	0.50	0.40	0.20	1.10	0.55 ± 0.10	0.30 ± 0.05
1.20	0.50	0.50	0.20	1.20	0.30 ± 0.10	0.15 ± 0.02

^a^*p* = 0.05, statistical significance calculated by one way ANOVA and obtained from three replicate experiments.

**Figure 1:**
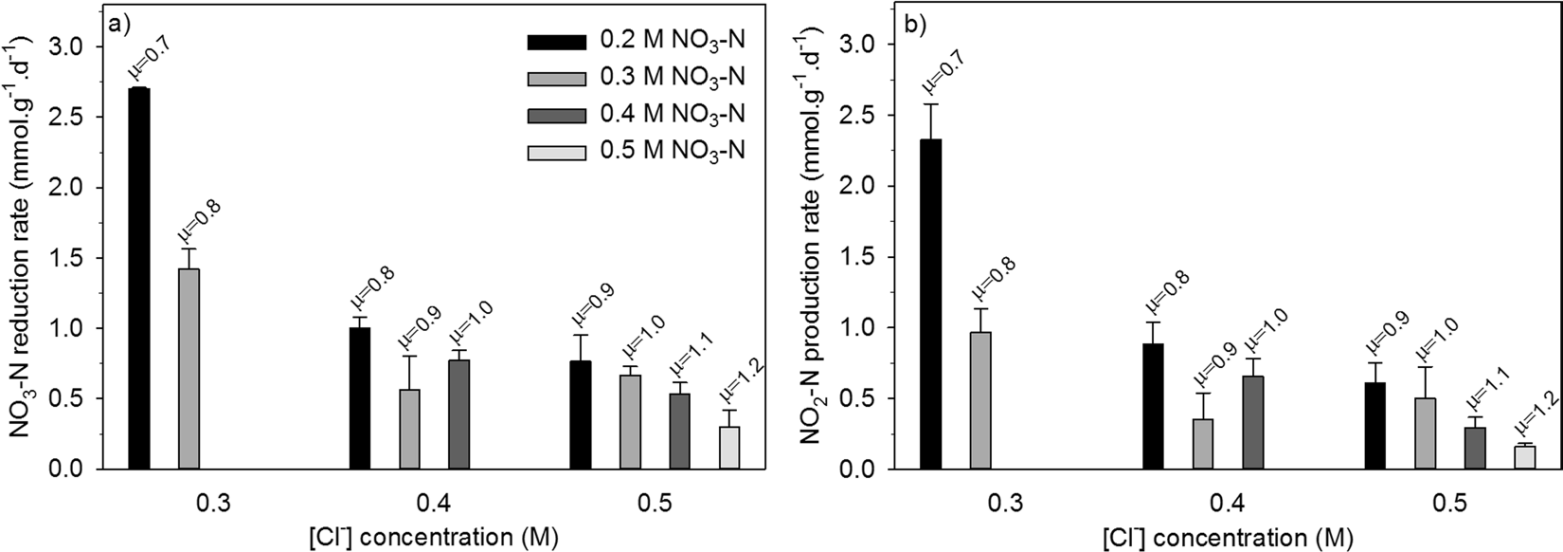
The influence of ionic activity and different [Cl^−^], [NO_3_^−^] concentrations on NO_3_^−^ reduction rate (**a**), and NO_2_^−^ production rate (**b**), of ACDC culture. Error bars represent standard deviation and statistical significance was determined by one way ANOVA (*n* = 3, *p* < 0.05).

The increase in either NO_3_^−^ or Cl^−^ ions caused a decrease in NO_3_^−^ reduction and NO_2_^−^ accumulation rates (Table 1 and Fig. 1). When different concentration couples were analysed it was found that the major reason behind the observed changes was the ionic strength (*µ*) of the solution rather than a change in specific ion concentration. Among the investigated concentration couples at similar ionic strengths NO_3_^−^ reduction and NO_2_^−^ accumulation rates were similar. For instance, in two cases where concentrations of the individual ions were different but the ionic activity of the solution was identical (*µ* = 0.8), the NO_3_^−^ reduction and NO_2_^−^ accumulation activity of ACDC was also similar and recorded as 1 mmol/g^−1^.d^−1^ (Fig. 1). Similarly, at *µ* = 0.9, the change in NO_3_^−^ reduction and NO_2_^−^ accumulation rates due to the change in concentrations of specific ions was not significant (*p* < 0.05). The negative influence of an increasing ionic strength was more pronounced between *µ* = 0.7 and *µ* = 0.9 when compared to the changes at higher ionic strengths (Fig. 1). At the ionic strength of a typical seawater (*µ* ~ 0.7 M), the NO_3_^−^ reduction and NO_2_^−^ accumulation rates were recorded as 2.70 mmol.g^−1^.d^−1^ and 2.30 mmol.g^−1^.d^−1^, respectively. These first results revealed that ACDC culture was adequate for the further tests in a corrosive chloride solution (0.5 M Cl^−^, *µ* = 0.5).

### Corrosion monitoring of the embedded steel rebars

The corrosion behaviour of rebars embedded in cracked MSCC was investigated by exposing the specimens to a 0.5 M Cl^−^ solution and measuring the open circuit potential (OCP) values for 120 days. The initial crack widths of the cracked mortar specimens were similar. The average crack widths were recorded as 310 ± 30 µm, 307 ± 41 µm, 348 ± 41 µm, 312 ± 23 µm and 300 ± 30 µm, for plain mortar, the positive control, the abiotic control, the self-healing control and the microbe-based test mortars, respectively.

**Figure 2:**
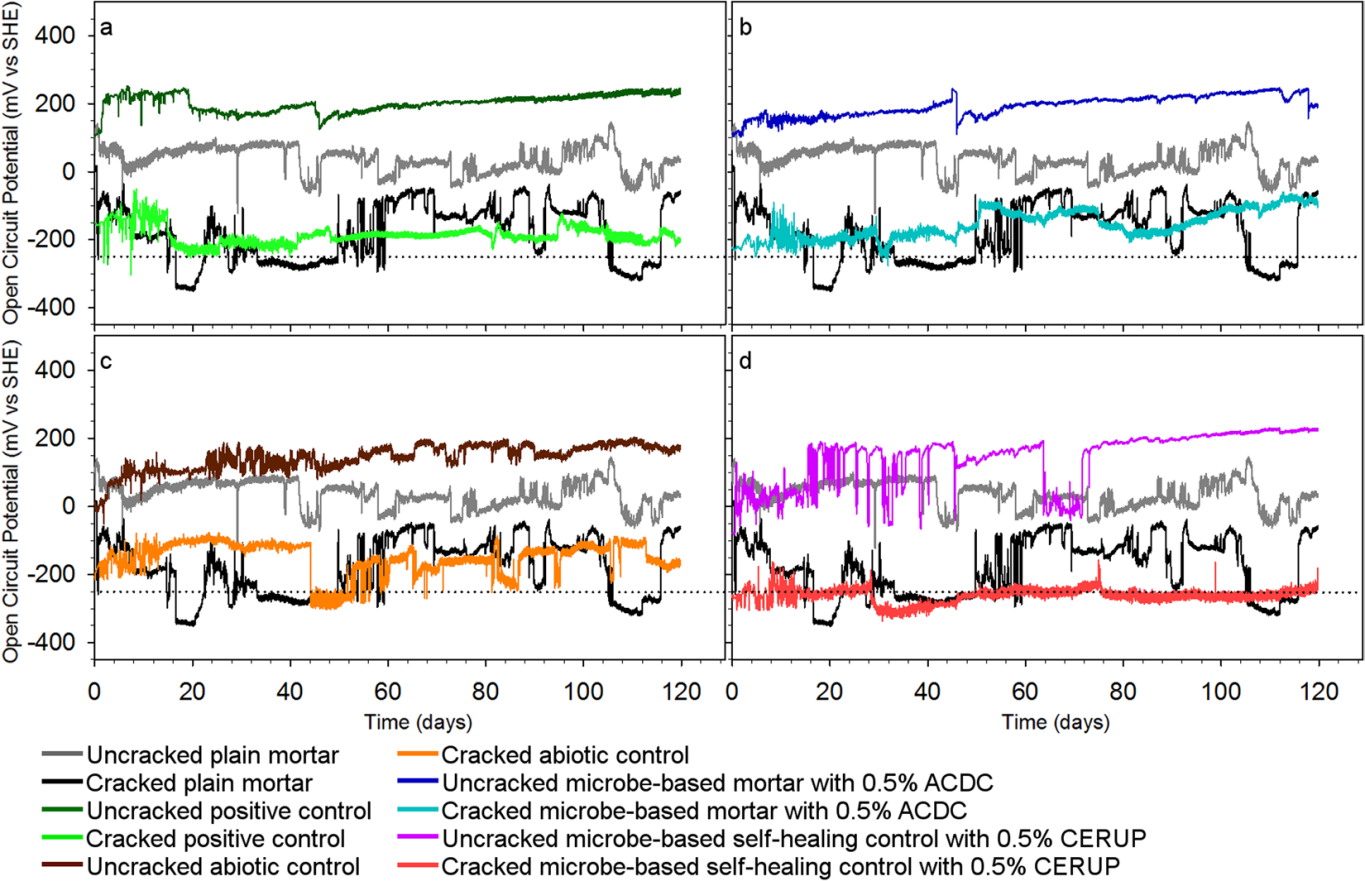
Evolution of OCP values of rebars embedded in different mortar series throughout 120 days exposure to 0.5 M Cl^−^ solution (**a**–**d**). Mean OCP values (*n* = 3) for rebars embedded in positive control mortar containing chemical NO_2_^−^ (**a**), the ACDC-based test mortar where biogenic conversion of NO_3_^−^ to NO_2_^−^ occurs (**b**), the abiotic control mortar initially containing NO_3_^−^ (**c**), the CERUP-based self-healing control mortar solely capable of microbe-mediated crack healing (**d**), are shown together with the values obtained in plain mortar. The dashed horizontal line (···) indicates the critical OCP value (−250 mV) for Fe in the Cl^−^ solution at a given pH and DO^28^.

The pH of the Cl^−^ solution showed minor variations and was recorded as 9.1 ± 0.1 over the course of the experiments (Supplementary Fig. S1) and the dissolved oxygen concentrations were recorded as 7.3 ± 0.2 mg/L. Considering the recorded pH value, dissolved oxygen concentration and the Cl^−^ concentration of the solution, the critical OCP value for possible steel corrosion was determined as ~−250 mV (versus that of a standard hydrogen electrode, or SHE) (Supplementary Fig. S2). Figure 2 presents the OCP values of the rebars embedded in different cracked and uncracked mortar specimens during exposure to 0.5 M Cl^−^ solution. In order to distinguish the effect of the mortar type on corrosion behaviour of the embedded rebars, OCP values obtained in different types of mortars were presented together with the OCP values of rebars in plain mortar (Fig. 2a–d). The OCP results revealed that rebar corrosion initiated in the cracked plain mortar after 16 days exposure to 0.5 M Cl^−^ solution (significant drop below −250 mV) while the rebar embedded in positive control specimens containing NO_2_^−^ always showed OCP values higher than −250 mV throughout the 120-days experimental period (Fig. 2a). The OCP values of the rebar embedded in the mortar specimens containing ACDC were also stable above −250 mV (Fig. 2b). In both mortar types, no sharp decrease occurred in the OCP value throughout the test. On the other hand, the rebar in the abiotic control mortar started corroding after 44 days (Fig. 2c). The rebars in the self-healing control started to corrode on day 28 (Fig. 2d). In all corrosion cases, following corrosion initiation, the OCP values dropped sharply and repetitively to/below the defined critical value (−250 mV) indicating an ongoing corrosion.

### Effect of crack healing on rebar corrosion

**Figure 3:**
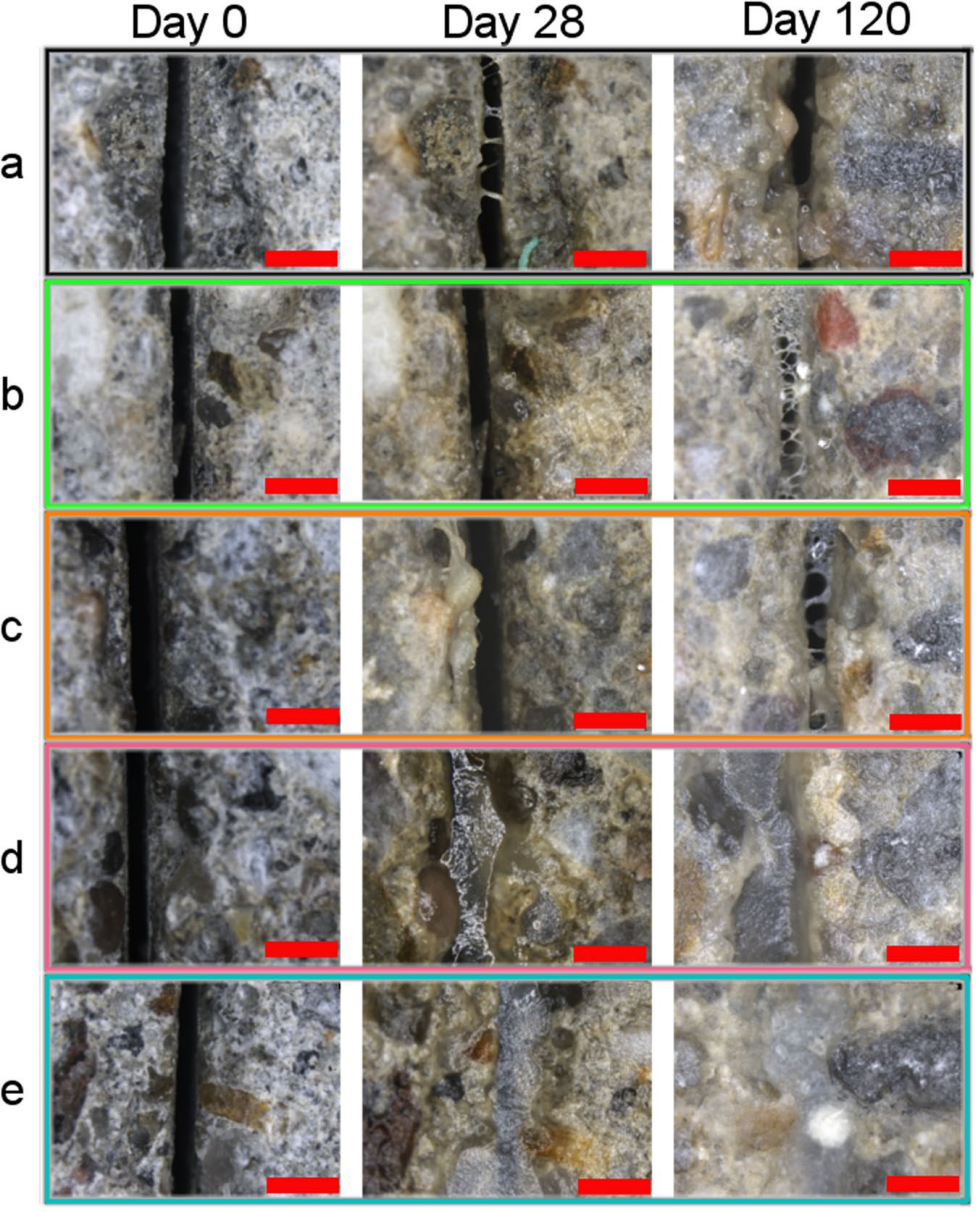
Self-healing of cracks in different mortar series. (**a**–**e**) Representative micrographs showing the evolution of the 300-µm-wide cracks in 120 days in plain mortar control (**a**), positive control mortar containing chemical NO_2_^−^ (**b**), the abiotic control mortar initially containing NO_3_^−^ (**c**), the CERUP-based self-healing control mortar solely capable of microbe-mediated crack healing (**d**), the ACDC-based test mortar where biogenic conversion of NO_3_^−^ to NO_2_^−^ occurs (**e**). Scale bars, 1 mm.

Among the cracked mortar specimens, only microbe-based mortars showed complete healing of the cracks in a short period of time (Fig. 3). Mortar containing ACDC granules showed complete crack closure in 28 days similar to the self-healing control mortar containing ureolytic bacteria (Fig. 3d,e). The healing material was stable in both mortars until the end of the experiments (Fig. 3d,e). However, upon crack closure (between day 28 and 120), the rebar protection performance of the self-healing control mortar did not become any better than that of the other negative controls (i.e. abiotic control and plain mortar) (Fig. 2c,d).

### Gravimetric, visual and chemical analyses of the extracted rebars

**Figure 4:**
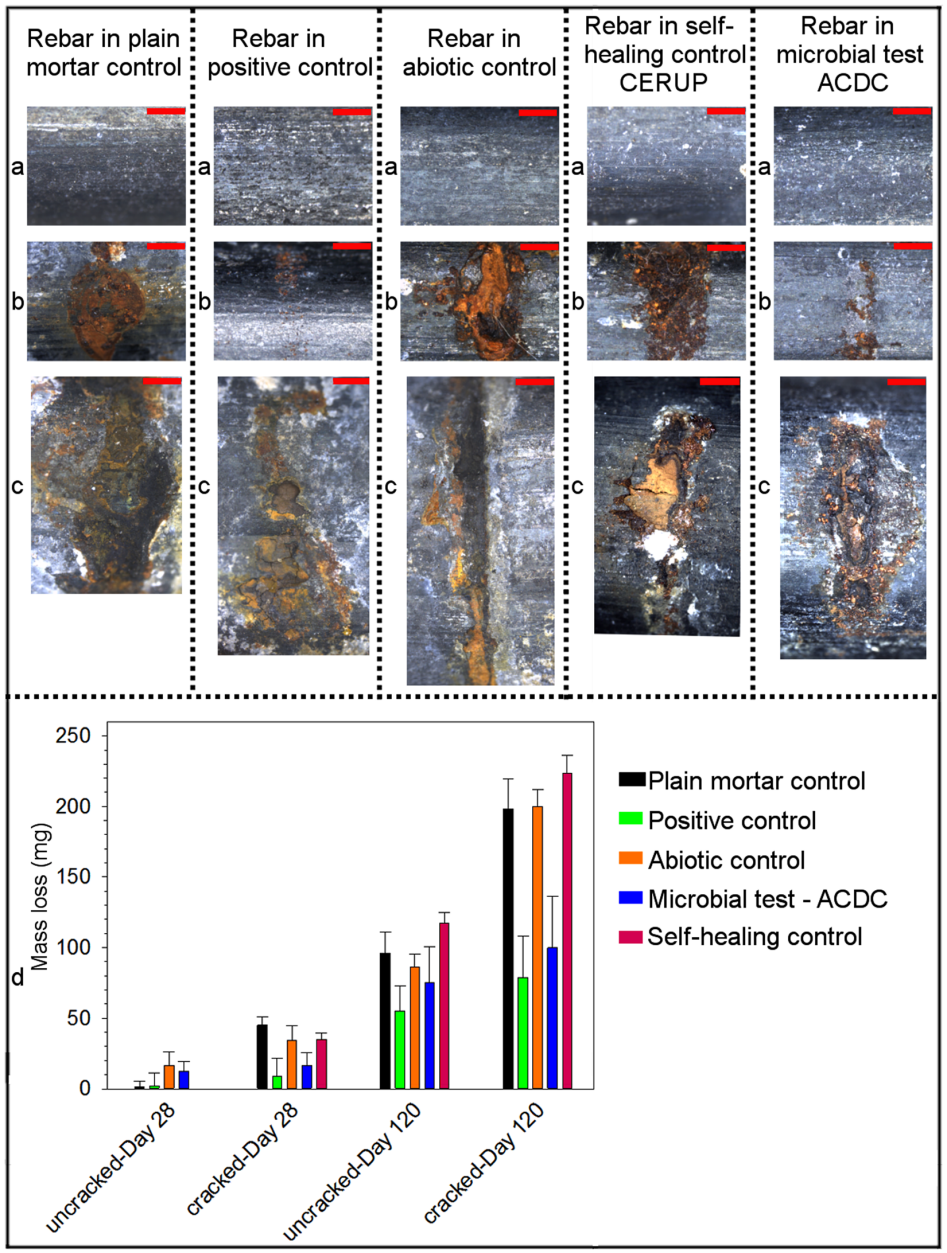
Influence of corrosion on surface appearances and masses of the rebars. (**a**–**c**) Representative micrographs showing the surfaces of the rebars taken from the indicated uncracked mortar series at the end of the 120-day testing period (**a**), from cracked mortar series at the end of 28-day testing period (**b**), from cracked mortar series at the end of 120-day testing period (**c**). Scale bars, 1 mm. (**d**) The quantified gravimetric mass loss due to corrosion (*n* = 3, *p* < 0.05). Error bars represent standard deviation and statistical significance was determined by one way ANOVA.

The gravimetric mass loss of the rebars due to corrosion was quantified and the alterations in their surface properties were examined. Regardless of the mortar type, the rebars taken out from the uncracked mortars at the end of 120 days exposure to corrosive solution had an intact surface without any rust formation (Fig. 4a). Among different cracked mortar specimens, the rebars taken out from the positive control mortar and the ACDC containing mortar exhibited a remarkably intact surface at the end of the pre-healing period (Fig. 4b) which correlated well with the aforementioned OCP results. After long-term (120 days) exposure to salt solution, the obtained micrographs became insufficient to distinguish the surface degradation of rebars of different series (Fig. 4c). Nevertheless, the mass losses showed that ACDC containing mortar could reach the rebar protection performance of uncracked mortar in both pre- and post-healing periods, similar to the positive control (*p* < 0.05) (Fig. 4d). For the rebars embedded in ACDC containing mortars, the mass loss corresponded to 50% of the loss recorded for negative control specimens (Fig. 4d). When corrosion inhibition occurred, the rebars lost less than 2% of the total material (average rebar mass 5.5 ± 0.2 g, *n* = 30) at the end of 120 days exposure to salt solution (Fig. 4d).

**Figure 5:**
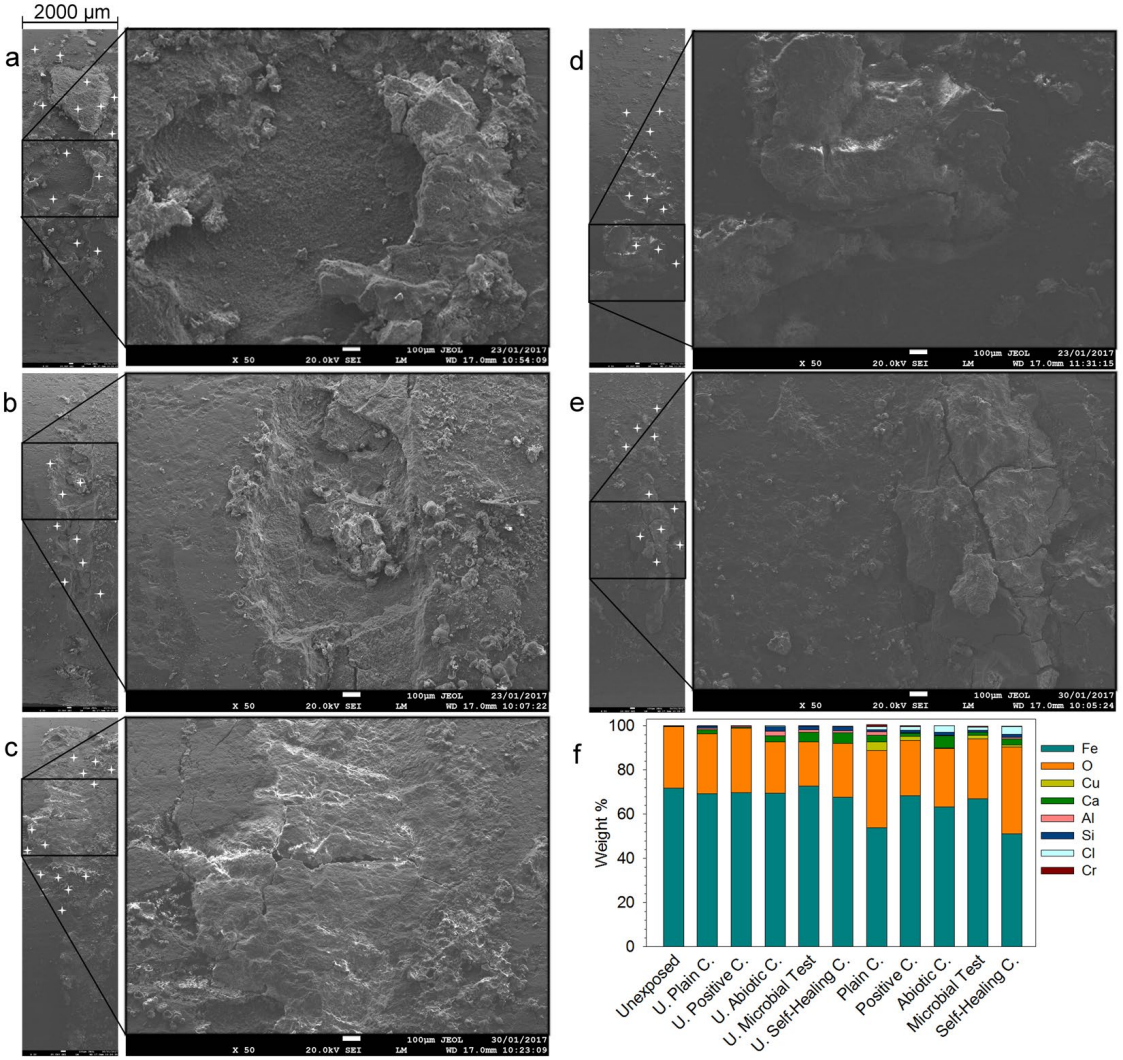
Impact of 120 days of exposure to Cl^−^ solution on integrity of the passive oxide layer and surface composition. (**a**–**e**) Representative low-magnification (left) and high-magnification (right) SEM micrographs (2 mm wide) of the rebar surfaces after 120 days of exposure to the Cl^−^ solution, in cracked plain mortar (**a**), in cracked abiotic control mortar (**b**), in cracked self-healing control mortar (**c**), in cracked positive control mortar (**d**), and in cracked microbial test mortar (**e**). The white crosses indicate the points where the EDS analyses were performed. White scale bars are 0.1 mm. (**f**) Abundant elements found on the surface of the rebars via EDS analysis (*n* = 8), U: uncracked, C: control (see Supplementary Fig. S3 for SEM micrographs and EDS sampling of uncracked specimens).

Scanning electron microscope (SEM) micrographs of the rebar surfaces were acquired and energy dispersive spectroscopy (EDS) analyses were conducted to clearly distinguish between the surface degradation of rebars taken from different mortars after 120 days (Fig. 5). A large cavity was spotted on the surface of the rebar taken from the plain mortar (Fig. 5a). Similarly, cavities and pits were observed on the surface of the rebars taken from the other negative controls (Fig. 5b,c). The corrosion spread around the exposed crack zone was the common observation for all the rebars taken from negative control mortars (Fig. 5a–c). The surfaces of the rebars that were taken from the positive control mortars and mortars with ACDC were free from uniform corrosion (loss in thickness) (Fig. 5d,e). Some clusters of rust were observed on the area which was exposed to the corrosive solution for 120 days. These distinct differences in corrosion behaviour of the samples were consistent with the abovementioned mass loss results.

EDS analyses were conducted to identify the type of material lost from the rebars due to corrosion. The surface composition of an unexposed rebar was 71 ± 1% Fe and 27 ± 1% O (*n*_EDS_ = 9) which indicated that the surface was mostly composed of iron oxides (Fig. 5f). Compared to a typical unexposed rebar, the iron-oxide (Fe_x_O_y_) weight ratio, Fe content in particular, decreased in the corroded rebar surface as the corrosion became more severe, and other metals of the rebar alloy such as copper, chromium and aluminium appeared owing to the degraded passive layer (Fig. 5f). The surface of the rebar sample taken from ACDC containing cracked mortar, similar to the cracked positive control, showed 6% (wt/wt) decrease in iron-oxide content (*n*_EDS_ = 10) while in negative controls it was 10% (*n*_EDS_ = 13) (Fig. 5f).

## Discussion

In this study, we revealed that ACDC can be useful for concrete applications in marine environment. The culture can resuscitate and reduce NO_3_^−^ in aquatic environments with an ionic strength up to 1.2, which are essential metabolic activities to induce calcium carbonate precipitation and corrosion inhibition. At the ionic strength of a typical seawater (*µ* ~ 0.7 M), the specific NO_3_^−^ reduction and NO_2_^−^ accumulation rates of ACDC were found to be 2.7 mmol.g^−1^.d^−1^ and 2.3 mmol.g^−1^.d^−1^, respectively. Previous studies aiming at wastewater treatment reported NO_3_^−^ reduction rates as high as 86 mmol.g^−1^.d^−1^ (at *µ* = 0.8) after acclimating the nitrate reducing culture to high Cl^−^ and NO_3_^−^ concentrations^22^. It should be noted that the bacteria in those studies were already active, the composition was rich in nutrients (in terms of micro- and macro-nutrients) and considerable amount of time was spent for acclimation at high ionic strengths. In this study, only an electron donor (HCOO^−^) was available as macro-nutrient and the solution was deficient in terms of phosphorus and micro-nutrients. Moreover, the activity was recorded immediately after resuscitation of the ACDC culture without any acclimation. Therefore, the observed difference with the previously reported performances could be attributed to aforementioned harsh conditions in our test setup. The main objective of this first set of experiments was to clarify whether ACDC can resuscitate in marine conditions, reduce NO_3_^−^ and accumulate NO_2_^−^ by only relying on a readily degradable electron donor (HCOO^−^). It can be said that there is still room for improvement of the resuscitation performance and activity by making necessary modification on the cultivation process of ACDC. Perhaps such improvements can positively influence the corrosion inhibition performance as well, and thus should be noted for a further study.

The corrosion equally progressed in all cracked mortars without corrosion inhibitors (Fig. 4c). The delay in the corrosion initiation of abiotic control was due to the presence of NO_3_^−^, which is believed to have some inhibitory effect on the corrosion of steel^23^. However, the passivation due to NO_3_^−^ was not as effective as NO_2_^−^ (Fig. 4a,c) which was consistent with the previously reported results^3^, and thus significant protection could only be achieved in series containing NO_2_^−^ or by microbial conversion of NO_3_^−^ to NO_2_^−^. As the use of Ca(NO_3_)_2_ is economically more feasible than the use of Ca(NO_2_)_2_ (refs^2,3^), ACDC containing MSCC not only offers a bio-based alternative for corrosion prevention but it can also decrease the cost of rebar protection in marine environment.

The self-healing control tested in this study was significant to reveal the sole effect of microbial induced crack healing on the corrosion behaviour of the steel. Obtained results revealed that only biomineralization is not an effective method for protection of the steel reinforcement as opposed to the claims in previous microbe-based self-healing studies^4–6,9,14^. Corrosion of the steel reinforcement in self-healing control continued even after the noted complete crack closure on day 28. Although the crack closure performances of the two different types of MSCC tested in this study were similar here and reported to be similar earlier^6,7^, when they were compared in terms of steel protection, the one containing NO_3_^−^ reducing ACDC culture became more prominent owing to its remarkable corrosion prevention performance. The quality of the steel protection in ACDC containing MSCC was equivalent to that in the cracked positive control and that in uncracked mortar. Additionally, the closing of the crack in 28 days improved the aesthetic and functional properties of the ACDC containing specimen as compared to those of the positive control.

## Conclusion

Taken together, these findings unveiled the necessity of a corrosion inhibition mechanism together with the self-healing functionality to delay rebar corrosion in cracked cementitious composites and to limit its development in the post-healing period. By using NO_3_^−^ reducing ACDC culture, we developed an advanced MSCC that can protect the rebar in cracked mortar up to 4 months while healing a 300 µm wide crack in the first 28 days. Corrosion inhibition achieved through microbial nitrate reduction was as effective as that achieved by using commercially used chemical inhibitors such as Ca(NO_2_)_2_. The material performance is promising to effectively protect the rebar against corrosion in marine environment and increase the service life of structures. We expect such composites to be applicable in a broad range of environments including underwater tunnels and nuclear repositories. Our advanced approach may also contribute to other concepts in microbial induced corrosion inhibition such as bio-competitive exclusion. Overall, these represent a departure from certain existing chemical based corrosion inhibitors which are not welcomed in the scope of a more sustainable world.

## Materials and Methods

### Microbial cultures and biomineralization precursors

Microbial NO_3_^−^ reduction was investigated by using a non-axenic self-protected NO_3_^−^ reducing culture called ACDC. A cylindrical sequencing batch reactor (SBR) was operated under feast/famine conditions for four months (4 cycles/day) by slightly modifying a previously described operational procedure^7^ and in total 35 g of ACDC granules were cultivated. Different from the previous setup, height to diameter ratio of the SBR was 10 (effective *h* = 80 cm, Ø = 8 cm). Minimal nutrient solution (COD: N—5:1) was used as feed (4 times/day) and the solution composition is given in Table S1. Initial pH of the synthetic feed solution was set to 9.5 by using concentrated NaOH solution (10 M). Full granulation (94% of the volatile suspended solid, VSS content) was achieved in 5 weeks and the first granules were harvested in the 6^th^ week of the operation. Further time points for harvests were arbitrarily chosen after achieving the stable granular biomass (94% of the biomass) with an active denitrifying core. The harvested ACDC granules were dried for 36 h in a drying tunnel at 60 °C with ventilation and stored at room temperature until their incorporation into the mortar specimens. The VSS content (which represents the amount of bacteria and organic matter inside the granular culture) was 70% of the ‘total suspended solids’ (TSS) content (which represents the sum of the bacteria and organic matter and the inorganic salts) of the granulated culture.

In corrosion experiments, as a control culture, we used a self-protected non-axenic ureolytic culture, so called CERUP, so its effect on mortar properties is similar to ACDC and it can induce crack healing through urea hydrolysis^6,24^. The CERUP used in this study was cultivated in Avecom, NV, Belgium based on the previously described thermo-cycle based procedure that induce sporulation (at 70 °C) twice a day^6^. The VSS content of the obtained CERUP culture was 50% of the TSS content. The feed composition used in CERUP production is given in Table S1.

Admixtures of Ca(HCOO)_2_ and Ca(NO_3_)_2_ were used as biomineralization precursors (details are given for each batch in the following section) for the ACDC culture, and CO(NH_2_)_2_ was used as a biomineralization precursor for the CERUP culture, in the mortar.

### Assessment of the tolerance of microbial culture to osmotic stress and ionic strength

ACDC culture was tested at varying ionic strengths by changing the initial Cl^−^ and NO_3_^−^ concentrations. The test conditions are given in Table 1. Mixtures in batch reactors were prepared by using sterile ultra-pure water, a fixed amount of a carbon source (0.2 M NaHCOO) and combinations of Cl^−^ and NO_3_^−^ concentrations. We chose a Cl^−^ concentration range from 0.3 M (which has no effect)^25^ to 0.5 M (that of typical seawater), in 0.1 M increments. The tested NO_3_^−^ concentrations were determined on the basis of the Cl^−^ concentrations. The NO_3_^−^ concentrations we chose ensured a [NO_2_^−^]:[Cl^−^] ratio of at least 0.34:1.00 when NO_3_^−^ was completely reduced to NO_2_^−^. This ratio was previously found to be the minimum ratio for effective steel corrosion inhibition in concrete environment^15^. The initial pH inside the batch reactors was set to 9.5 by using NaOH (1 M). The initial VSS concentration in the reactors was 2.5 g/L. Abiotic controls were also tested for each mixture.

### Experimental planning of corrosion tests and investigated mortar series

In the experimental design, in order to distinguish the anodic corrosion inhibition occurring due to microbially produced NO_2_^−^, four control series were investigated in addition to the microbial test mortar containing ACDC granules. The control series were (i) a plain mortar, (ii) a positive control (sole effect of chemical NO_2_^−^), (iii) an abiotic control (the effect of nutrients) and (iv) a microbe-based self-healing control (the sole effect of crack closure due to biomineralization induced by previously reported non-axenic cyclic enriched ureolytic powder, or CERUP^6^, without NO_2_^−^ production). The plain mortar series contained DIN EN 196-1 standard sand (1350 g), CEM I 52.5 N cement (450 g) and tap water (225 g), with 3.0:1.0:0.5 wt/wt ratios according to the EN 196-1 standard. Microbial test mortar contained ACDC-VSS (0.5% wt/wt cement), Ca(NO_3_)_2_ (3% wt/wt cement) and Ca(HCOO)_2_ (2% wt/wt cement) in addition to the components present in plain mortar. The positive control mortar contained NO_2_^−^ (1.6% wt/wt cement) as a corrosion inhibitor in the form of NaNO_2_ (2.4% wt/wt cement) and Ca(HCOO)_2_ (2% wt/wt cement). Abiotic control mortar contained the nutrients of the microbe-based test batch, Ca(NO_3_)_2_ (3% wt/wt cement) and Ca(HCOO)_2_ (2% wt/wt cement). The microbial self-healing control mortar contained CERUP-VSS (0.5% wt/wt cement) and CO(NH_2_)_2_ (5% wt/wt cement). The amount of nutrients in microbe-based mortars were determined based on the previous studies where successful crack healing performances were achieved by means of ACDC and CERUP^6,7^.

Each series was tested under ‘uncracked’ and ‘artificially cracked’ conditions. In each series 12 specimens were tested (*n*_*cracked*_ = 6, *n*_*uncracked*_ = 6). Half of each series was monitored for 28 days and the other half was monitored for 120 days. We defined the first 28 days of the Cl^−^ exposure experiment as pre-healing period on the basis of the previous self-healing reports on MSCC^6,7^. The results obtained for the 28-days-tested specimens (*n*_*cracked*_ = 3, *n*_*uncracked*_ = 3) were analysed to determine the corrosion behaviour of rebars embedded in different mortars with 300 µm wide cracks and the influence of microbe-mediated self-healing on corrosion behaviour. The other half of the mortar specimens (*n*_*cracked*_ = 3, *n*_*uncracked*_ = 3) were monitored further in Cl^−^ solution, until day 120. Results obtained throughout 120 days exposure were interpreted to evaluate the corrosion behaviour of a microbial self-healing reinforced mortar in an extended time period subsequent to crack healing (combination of pre-healing and post-healing exposure to salt solution).

### Preparation of the mortar specimens and formation of the cracks

By following the procedure in the EN 196-1 standard, a series of mortar specimens (40 mm × 40 mm × 160 mm) with a centrally positioned embedded smooth steel rebar (NBN A-24 302 BE 220 S Grade, with a nominal diameter of Ø = 8 mm) were prepared.

During mortar preparation all of the admixtures and bacteria were added in powdered form to the mixture, except CO(NH_2_)_2_ which was dissolved in the mixing water prior to addition. Bacterial admixtures, ACDC and CERUP, were 0.5–2 mm in size.

Artificial cracks were created for testing, because the creation of realistic cracks would affect the reproducibility of the results and complicate the comparison of different series. The cracks were formed according to the procedure described previously^26^, by using 300 µm thick brass plates with a semi-circular notch (Ø = 8 mm). The plates were positioned with their notch partially surrounding the steel rebar inside the moulds, and thus, it was assured that the crack reached and partially surrounded (upper half of the circumference) the steel rebar. After arranging the plate positions to obtain three cracked and three uncracked specimens from each moulded prism, the mortar was poured. After curing the specimens for 24 hours at a temperature of 20 °C and a relative humidity (RH) of >90%, the plates were carefully removed. This resulted in the formation of artificial cracks with a depth of 20 mm and a width of ~300 µm over the complete 40-mm width of the mortar samples.

At the end of the 28-days curing period at a temperature of 20 °C and a relative humidity (RH) of >90%, the trowelled surface of the mortar prisms was polished (removing a 2 mm thick layer) to create a uniform flat test surface. Subsequently, six artificially cracked and six uncracked pieces (38 mm × 40 mm × 15 mm) were sawn off from the prisms via wet sawing.

The widths of the artificially created cracks were confirmed with a stereomicroscope (Leica s8 APO with a DFC 295 camera) (Diegem, Belgium). At the end of the 28-days and 120-days experimental periods, the crack widths were measured again to quantify self-healing performance.

### Corrosion testing

Capillary sorption was used to create the conditions for corrosion. The cracked surfaces of the specimens were soaked with 0.5 M Cl^−^ solution and kept in contact with the solution for 28 and 120 days. The exposed surfaces were standardized (40 mm × 15 mm) by partially covering the side surface area of the mortar samples with aluminium butyl tape. Capillary sorption occurred only through the pores and cracks on the cracked surface that was in contact with the solution. Throughout the experiments, the dissolved oxygen concentration in the bulk solutions was 7.3 ± 0.2 mg/L and the pH was 9.1 ± 0.1.

Identical conductive, insulated copper wires were placed in contact with one end of the steel rebar to create an electrical connection between the steel rebar and the data logging system. The wires were fixed to the steel rebar and covered with a non-conductive patch. To monitor the electrochemical corrosion potential, we used a data-logging system which recorded the open circuit potential (OCP) versus a reference saturated calomel electrode (SCE) for 120 days. Different batches were separated into different containers, and specimens were placed at similar distances from the reference SCE. Data were recorded every 6 min. For the first 28 days, the validity of the automated system was checked every week through additional separate OCP measurements in equimolar Cl^−^ solution (0.5 M Cl^−^) by using a Gamry Interface 1000 potentiostat (Gamry Instruments, USA). The setup parameters for the weekly OCP measurements were 500 sec, 0.5 sec and 0 mV/sec for the total measuring time, data sampling period and stability, respectively. All of the obtained data (versus reference SCE) were converted to values versus the standard hydrogen electrode (SHE), on the basis of the given equilibrium potentials^27^, to be able to interpret them using the Pourbaix diagram of the iron species. Obtained data was compared with the limit OCP value (−250 mV vs SHE) defined based on the findings in Moreno *et al*.^28^ (Supplementary Fig. S2) and also compared with the OCP values defined in ASTM C876-91 for different corrosion probabilities^29^ (Supplementary Table S2).

### Gravimetric loss analysis

Pieces of steel rebar of varying lengths (from 14 to 18 mm) were sawn off from a smooth unexposed steel rebar (NBN A-24 302 BE 220S Grade, with a nominal diameter of Ø = 8 mm), and their average density values were calculated. The lengths of the tested steel rebars (which were embedded in the mortar specimens) were accurately measured, using an electronic calliper, before the corrosion experiments. Therefore, the initial volume of each embedded rebar could be calculated. The initial mass was then calculated by using the average density of the steel rebar and the initial volume. At the end of the experiments, the steel rebars were taken out of the tested specimens by breaking the mortar, and all corrosion products were removed by gentle cleaning under running water. After drying with compressed air they were weighed. The difference between the calculated initial mass and the measured final mass is presented as the gravimetric mass loss.

### Observation of corrosion under an optical and scanning electron microscope

The degree of corrosion at the position where the crack reached the steel rebar, as well as the general state of the steel rebar, was observed under a stereomicroscope using Leica S8 Apo apochromatic optics (Diegem, Belgium). The resulting micrographs were used to differentiate between the corroded specimens of different batches. For the series that had been exposed to a Cl^−^ solution for 120 days, further analyses were performed with a scanning electron microscope (SEM) (FEI Quanta 200 F SEM/EDX, Oregon, USA) coupled with energy-dispersive X-ray spectroscopy (EDS). SEM micrographs were taken at accelerating voltage of 20 kV at a working distance of 17 mm. EDS analysis was conducted on the surface of the unexposed steel rebar to determine the actual surface composition of the steel rebar in terms of Fe_x_O_y_ and Fe (wt%). Afterwards, the surface compositions of the exposed steel rebars were analysed. Reductions in Fe_x_O_y_ content (wt%) were used to distinguish the corrosion degree of rebars taken from different batches.

### Analytical methods

TSS and VSS analyses of the grown ACDC culture and obtained CERUP culture were conducted according to standard methods^30^. Throughout the experiments, the ion concentrations (NO_3_^−^, NO_2_^−^ and Cl^−^) were measured via compact Metrohm 930 ion chromatography (IC) (Herisau, Switzerland).

### Statistical analyses

Unless stated otherwise, all of the experiments were conducted in triplicate and the relevant data are presented as mean ± standard deviation. Mean values of OCP measurements are presented without giving the standard deviations as they negatively affect the visibility of the presented data which was recorded every 6 min for 120 days. Sigma Plot v12.0 (Systat Software Inc, USA) was used to compare the significant differences in values by means of a one-way analysis of variance (ANOVA) test (*p* < 0.05).

## Electronic supplementary material

Supplementary Materials
