# Supplementary material for: Nitrite producing bacteria inhibit reinforcement bar corrosion in cementitious materials
Source: Sci Rep. 2018 Sep 20;8:14092. doi: 10.1038/s41598-018-32463-6 (PMC6148264; doi:10.1038/s41598-018-32463-6)
Supplement: Supplementary file 1 — Supplementary Materials [file 41598_2018_32463_MOESM1_ESM.pdf]

SCIENTIFIC REPORTS

Supplementary Materials for

Nitrite producing bacteria inhibit reinforcement bar corrosion in cementitious materials

Yusuf Çağatay ERŞAN[1,2], Kim VAN TITTELBOOM[1], Nico BOON[2], Nele DE BELIE[1]*

Affiliations:
[1]Magnel Laboratory for Concrete Research, Faculty of Engineering and Architecture, Ghent University, B-9052, Ghent, Belgium.
[2]Centre for Microbial Ecology and Technology (CMET), Faculty of Bioscience Engineering, Ghent University, B-9000, Ghent, Belgium.
*nele.debelie@ugent.be

This document includes

Tables S1 to S2

Figures S1 to S3

Number of pages: 6

Table S1. Feed composition for production of ACDC and CERUP

**Compounds**	**Concentrations (g/L)**	
	**ACDC production**	**CERUP production**
**$NaNO_3$**	1.7	-
**NaHCOO**	5.36	-
**$Ca(HCOO)_2$**	0.65	-
**$Na_2HPO_4.2H_2O$**	0.06	-
**$MgSO_4$**	0.1	0.5
**$C_{12}H_{22}O_{11}$**	-	2
**$CO(NH_2)_2$**	-	10
**KH_2PO_4**		0.5

Table S2. Probability of corrosion activity as a function of ranges of E_{corr} versus Standard Hydrogen Electrode (redrafted after[31])

**Corrosion condition**	***E_{corr}* vs SHE(mV)**
Low probability (<10%)	> 118
Intermediate probability	118 to –32
High probability (>90%)	< –32
Severe corrosion	< –182

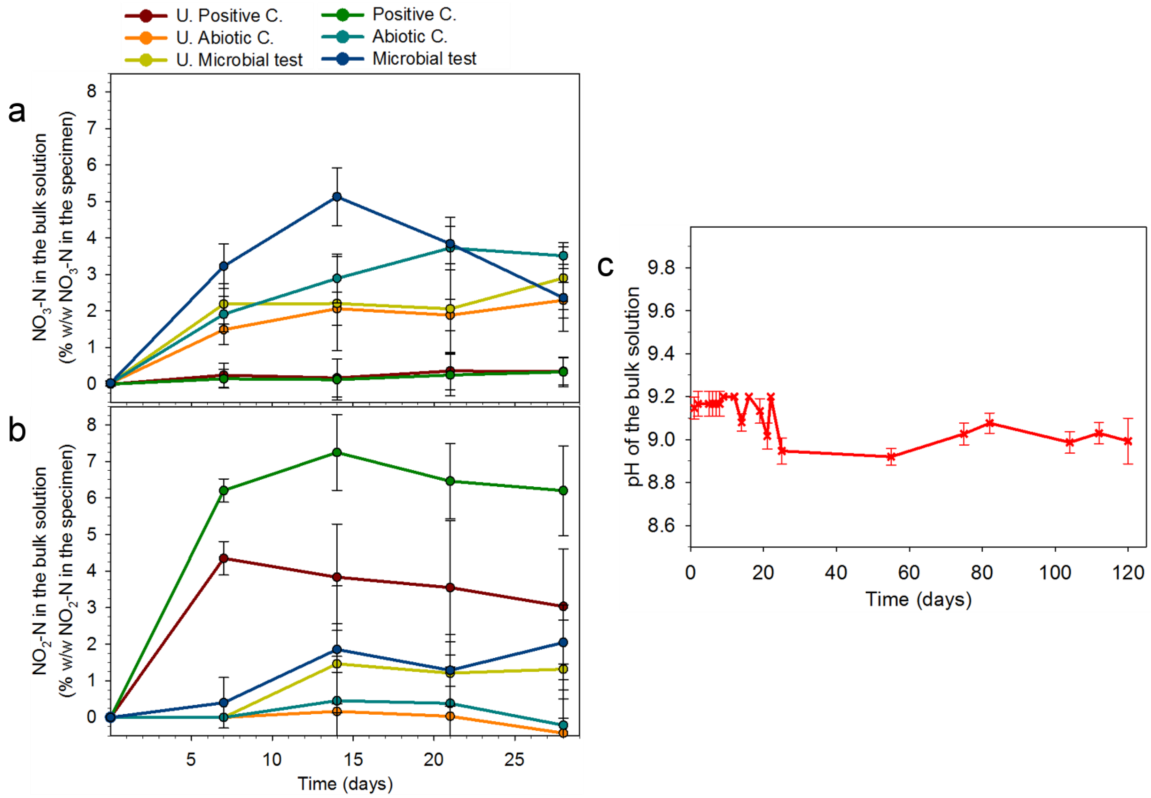

Figure S1. Microbial reduction of NO_3^- to NO_2^- that was traced through the bulk solution for 28 days and the change in pH over 120 days. (**A**) NO_3-N in the bulk solution that was leaching from NO_x–containing specimens (*n*=3), (**B**) NO_2-N in the bulk solution due to leaching from $NaNO_2$-containing specimens or due to the microbial conversion of NO_3-N leached from $Ca(NO_3)_2$-containing specimens (*n*=3); and (**C**) the average pH values observed in the bulk solution. The NO_x-N data are given in terms of the % wt/wt NO_x-N present in a single specimen, assuming the homogenous distribution of the admixtures. Theoretically, a single microbial specimen (38 mm × 40 mm × 15 mm) contains 45 mg NO_3-N and a single positive control specimen contains 45 mg NO_2-N. Error bars represent the standard deviation, p=0.05 statistical significance calculated by one way ANOVA and obtained from three replicate experiments.

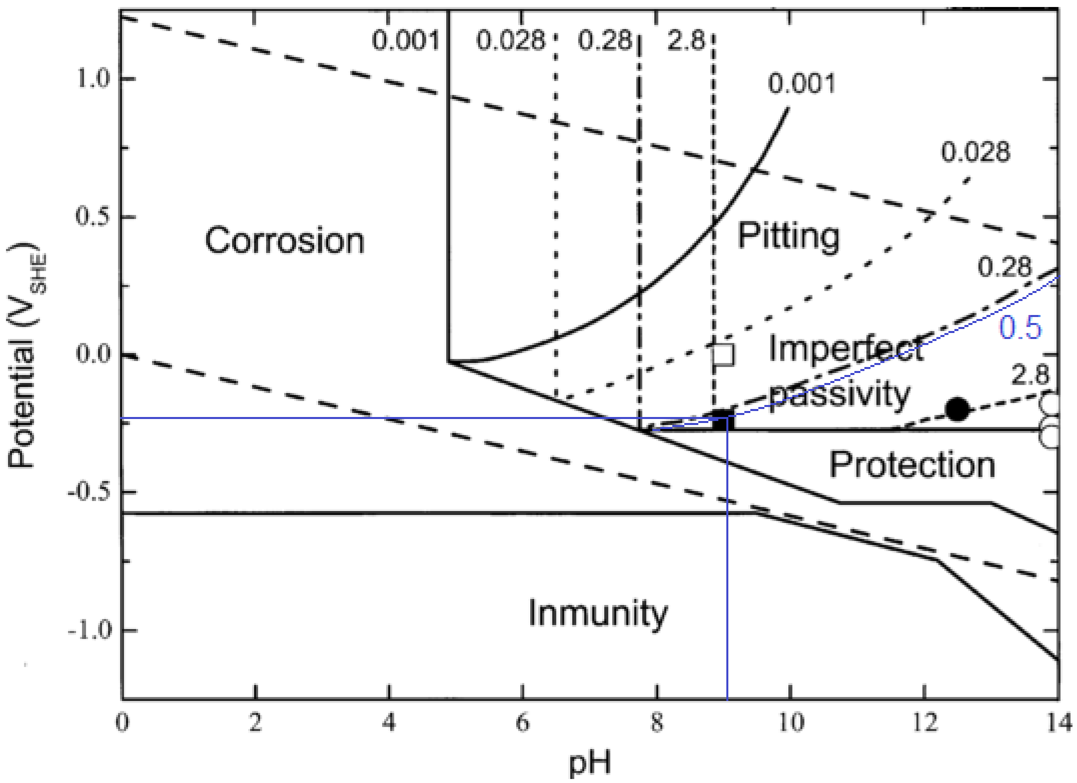

Figure S2. Severe rebar corrosion is favored when the OCP value decreases to less than –250 mV at presence of 0.5 M Cl^- at pH 9.1 ± 0.1, dissolved oxygen concentration of 7.3 ± 0.2 mg/L (redrafted after[23])

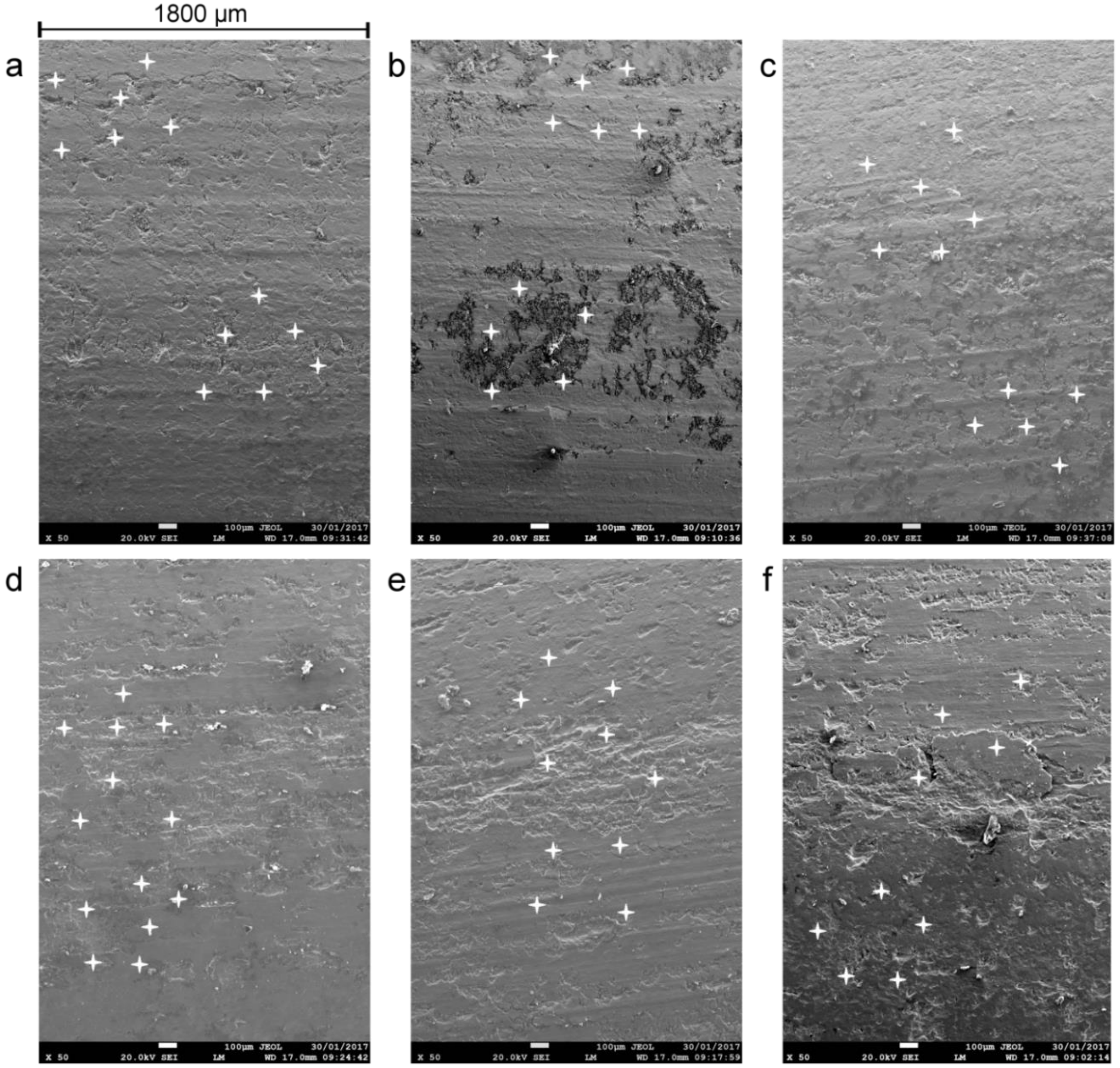

Figure S3. Representative SEM micrographs of the rebars taken from the uncracked mortar specimens after 120 days of exposure to Cl^{-} solution. (**A**) The plain mortar control, (**B**) the positive control, (**C**) the abiotic control, (**D**) the microbial test, (**E**) the self-healing control and (**F**) unexposed steel rebar. White marks indicate the points where EDS analyses were performed. The white scale bars in the SEM images are identical and indicate 0.1 mm.
